# A 94 GHz Millimeter-Wave Radar System for Remote Vehicle Height Measurement to Prevent Bridge Collisions

**DOI:** 10.3390/s26061921

**Published:** 2026-03-18

**Authors:** Natan Steinmetz, Eyal Magori, Yael Balal, Yonatan B. Sudai, Nezah Balal

**Affiliations:** 1 Department of Electrical and Electronics Engineering, Ariel University, Ariel 4076414, Israel; 2 Faculty of Engineering, Jerusalem College of Technology, Jerusalem 9116001, Israel; 3 Department of Electrical and Electronics Engineering, Afeka College of Engineering, Tel Aviv 6910717, Israel

**Keywords:** millimeter-wave radar, Doppler ratio, velocity-independent measurement, vehicle height detection, bridge collision prevention, W-band, intelligent transportation systems

## Abstract

Collisions between over-height vehicles and low-clearance bridges cause infrastructure damage and pose safety risks. Existing detection systems rely primarily on optical sensors, which suffer from performance degradation in adverse weather conditions. This paper presents an alternative approach based on a 94 GHz millimeter-wave radar that achieves velocity-independent height measurement. The proposed technique exploits the ratio of Doppler shifts from two scattering centers on a vehicle, specifically the roof and the wheel–road interface. This ratio depends only on the measurement geometry, as the unknown vehicle velocity cancels algebraically, enabling direct height computation without speed measurement. The paper provides a closed-form height estimation model, analyzes the trade-off between frequency resolution and geometric constancy during integration, and presents experimental validation using a scaled laboratory testbed. An optical tracking system is used solely for ground-truth validation in the laboratory and is not required for operational deployment. Results across six test cases with heights ranging from 20 cm to 46 cm demonstrate an average absolute error of 0.60 cm and relative errors below 3.3 percent. A scaling analysis for representative full-scale geometries indicates that at highway speeds of 80 km/h, integration times in the millisecond range (approximately 3–18 ms for representative 20–50 m measurement standoff) are feasible; warning distance can be extended independently by upstream radar placement. The expected advantage in fog, rain, and dust is based on established W-band propagation characteristics; dedicated adverse-weather and full field validation (including multipath, clutter, and multi-vehicle scenarios) remain future work.

## 1. Introduction

### 1.1. Background and Motivation

Collisions of over-height vehicles with bridges, tunnels, and overhead structures constitute a persistent problem in transportation networks, resulting in structural damage, vehicle destruction, traffic disruption, and occasionally loss of life. Despite regulatory efforts and technological countermeasures, these incidents continue to occur. In this study, vehicle height is defined as the vertical distance from the road surface to the highest scattering point (the roof), while clearance refers to the safe passage height under the bridge structure.

Statistics from several countries illustrate the scope of the problem. In the United Kingdom, railway bridge strikes occur approximately every five hours on average, with 1666 incidents reported from 1 April 2024 to 31 March 2025 and around £12 million in rail-delay/cancellation costs [1]. In the United States, over 15,000 bridge strikes occur annually [2]. Between 2014 and 2018, multiple fatalities were attributed to trucks striking overpasses [3].

**Figure 1:**
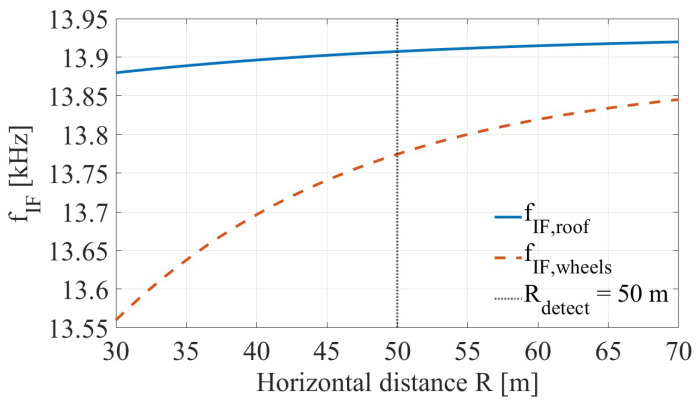
Theoretical intermediate-frequency (IF) Doppler components as a function of horizontal distance *R* for a truck traveling at v=80 km/h illuminated by a 94 GHz CW radar. The curves show the IF Doppler frequencies associated with the roof and wheel–road scattering centers for the nominal full-scale geometry. The vertical dotted line marks the design early-detection range $R_{\mathrm{detect}}=50$ m.

To address this challenge, an early-detection system is required. Figure 1 illustrates the theoretical Doppler frequency components for a truck traveling at 80 km/h as a function of distance from the radar. The distinct separation between the roof and wheel scattering centers, especially at the design detection range of R=50 m, provides the physical basis for the height estimation method proposed in this study.

**Figure 2:**
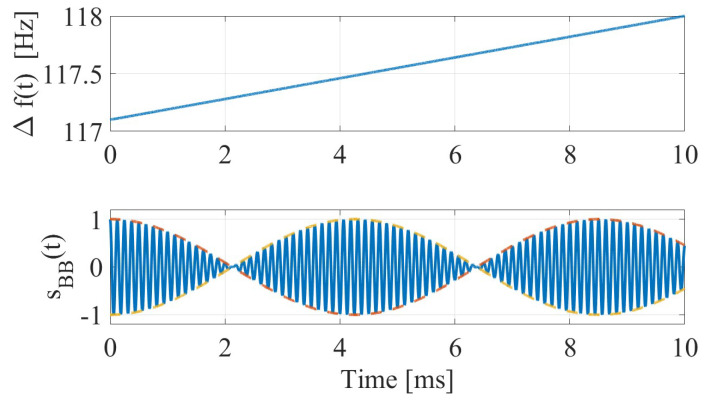
Theoreticalbeat-frequency behavior near the design detection range $R_{\mathrm{detect}}=50$ m for a truck traveling at v=80 km/h. (**Top**): instantaneous beat frequency $\Delta f\left(t\right)=f_{\mathrm{roof}}\left(t\right)-f_{\mathrm{wheels}}\left(t\right)$ over a 10 ms interval. (**Bottom**): synthesized baseband signal $s_{\mathrm{BB}}\left(t\right)$ with analytic envelope, illustrating the fast IF oscillation modulated by the slow beat frequency.

Repair costs typically range from £5000 to £25,000 in the UK and $200,000 to $300,000 in the United States [3,4], not including indirect costs such as traffic delays, emergency response, and cumulative structural degradation. The complexity of manual height estimation by drivers necessitates an automated sensing solution. The signal behavior for such a solution is depicted in Figure 2, which shows the beat-frequency characteristics near the 50 m detection range. As seen in the figure, the instantaneous frequency difference Δf(t) (top) and the resulting modulated baseband signal (bottom) allow for the extraction of height information independently of the vehicle’s absolute speed.

Studies consistently identify driver error as a primary factor. Research by Galer [5] found that only 12% of drivers accurately knew their vehicle height, and just 27% could estimate it within 76 mm. Contributing factors include unfamiliar routes, inadequate planning, unclear signage, and the growing number of tall vehicles [3]. The National Cooperative Highway Research Program has recognized this issue and recently published comprehensive guidance on mitigation strategies [2].

### 1.2. Current Detection Technologies

Existing over-height detection systems employ three main sensing modalities: infrared beam interruption, laser scanning (LiDAR), and camera-based computer vision. The most widely deployed approach uses dual-beam infrared sensors positioned at the clearance height; interruption by an over-height vehicle triggers warning devices [2]. These systems cost between $25,000 and $100,000 per installation and can reduce bridge strikes when properly deployed [4,6].

However, optical sensors share several fundamental limitations. Fog, rain, snow, and dust scatter and absorb optical wavelengths, degrading performance in adverse weather conditions [7]. Infrared systems can be falsely triggered by birds, debris, or vegetation [2]. Laser-based systems require careful calibration and may drift due to thermal expansion and vibration [8]. Direct sunlight can interfere with certain sensor configurations, and single-plane measurements may miss complex vehicle geometries. Computer vision approaches have shown promise [9,10] but remain sensitive to lighting conditions and weather, with reported false positive rates of approximately 0.2% under optimized conditions [10].

### 1.3. Millimeter-Wave Radar for Height Detection

Millimeter-wave (mmWave) radar at 94 GHz offers characteristics that address the limitations of optical sensors. Operating within an atmospheric transmission window in the W-band, radiation at this frequency propagates through fog, rain, snow, and dust with lower attenuation than optical wavelengths. Atmospheric attenuation at 94 GHz is approximately 0.4 dB/km in clear conditions and about 3 dB/km in moderate rain (4 mm/h) [11,12]. Studies demonstrate maintained radar operation in heavy fog with visibility as low as 0.5 m [11]. These propagation benefits motivate the proposed sensing modality; however, the present manuscript does not include dedicated fog/rain/wet-road experiments.

The short wavelength (λ≈3.2 mm) provides high Doppler sensitivity, enabling precise velocity-related measurements over short observation intervals [13]—a property central to the technique proposed here. The short wavelength also permits compact antennas with narrow beamwidths, and the technology is mature, with established applications in automotive radar [14], weather monitoring [15], security screening [16], biomedical sensing [17], and human activity recognition [18,19,20]. Our research group has previously demonstrated the effectiveness of 94 GHz micro-Doppler radar for applications including limb movement detection [18], fall detection [19], low-RCS target detection [21], and speech vibration characterization [20].

### 1.4. The Velocity-Independent Doppler Ratio Technique

Conventional Doppler radar measures radial velocity, which depends on both target speed and observation angle. Extracting height from a single Doppler measurement therefore requires independent knowledge of vehicle velocity, which is generally unavailable in infrastructure-based detection scenarios.

**Figure 3:**
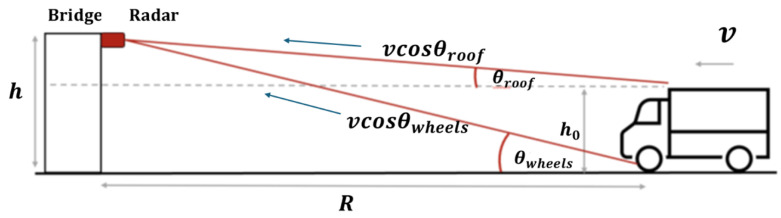
System geometry and velocity-cancellation interpretation for a monostatic 94 GHz CW radar. The projected terms *v* cos$\left(\theta_{\mathrm{roof}}\right)$ and *v* cos$\left(\theta_{\mathrm{wheels}}\right)$ generate the two Doppler components used in the ratio method.

The approach presented here addresses this limitation by analyzing the ratio of Doppler shifts from two distinct scattering centers on the vehicle: the roof and the wheel–road interface. The relevant system geometry is depicted in Figure 3, showing the radar at height *h*, the vehicle roof at height $h_{0}$, and the respective incidence angles $\theta_{\mathrm{roof}}$ and $\theta_{\mathrm{wheels}}$. Since both scattering centers move with the same horizontal velocity but subtend different angles to the radar, they produce different Doppler shifts. We define:

(1) $$\alpha =\frac{f_{\mathrm{roof}}}{f_{\mathrm{wheels}}}.$$

The explicit derivation of the angle-only form of α, which depends solely on geometry, is provided in Section 2 to avoid redundancy. Consequently, vehicle height can be computed directly from radar height, horizontal distance, and measured α without requiring velocity data.

Existing methods for height estimation often rely on optical sensors or specialized radar processing. While prior radar-based approaches have characterized vehicle height using profiles [22], FMCW ranging [23], or micro-Doppler signatures [24], these typically necessitate complex pattern recognition, extensive integration, or simulations to extract physical dimensions. The core innovation of the proposed technique is the algebraic ratio α, which enables the unknown vehicle velocity to cancel out completely within a closed-form equation. This allows for direct height computation without external speed measurement or computationally intensive processing, distinguishing this method from previous works. Furthermore, while vision-based methods [9] and modern LiDAR sensors offer high spatial resolution for object detection, their performance degrades significantly in adverse weather conditions such as fog, rain, or dust due to atmospheric scattering [25]. The proposed 94 GHz radar system therefore offers a physically motivated alternative; its deployment-level cost/accuracy advantages remain to be established through full-scale validation.

### 1.5. Contributions and Organization

This paper presents the development and experimental validation of a vehicle height measurement system using the velocity-independent Doppler ratio technique. The specific contributions include:

1. A closed-form height estimation model derived without small-angle approximations, together with a simplified analytical model for design guidance.
2. Comprehensive analysis of the trade-off between frequency resolution and geometric constancy as functions of integration time.
3. Experimental validation using a scaled laboratory testbed with optical ground truth, achieving an average absolute error of 0.60 cm across six test cases (20–46 cm heights).
4. A scaling analysis demonstrating that highway speeds yield larger Doppler shifts, enabling shorter integration times; field validation remains necessary.

The paper is organized as follows. Section 2 presents the theoretical framework. Section 3 describes the experimental system. Section 4 details signal processing methodology. Section 5 presents experimental results. Section 6 analyzes scalability to real-world conditions. Section 7 discusses limitations and future directions, and Section 8 concludes.

## 2. Theoretical Mathematical Modeling

The proposed system exploits the fundamental dependence of Doppler shift on both target velocity and incidence angle.

### 2.1. Geometric Configuration

Consider a monostatic radar mounted at height *h* above the road surface, illuminating a vehicle approaching at horizontal distance *R* with velocity *v*. We define two scattering centers: the roof at height $h_{0}$ and the wheels at ground level. The system geometry is shown in Figure 3.

The slant ranges from the radar to each scattering center are:

(2) $$r_{\mathrm{roof}}=\sqrt{R^{2}+{\left(h-h_{0}\right)}^{2}},\;r_{\mathrm{wheels}}=\sqrt{R^{2}+h^{2}},$$

and the corresponding incidence angles (measured from horizontal) satisfy:

(3) $$\cos\left(\theta_{\mathrm{roof}}\right)=\frac{R}{r_{\mathrm{roof}}},\;\cos\left(\theta_{\mathrm{wheels}}\right)=\frac{R}{r_{\mathrm{wheels}}}.$$

### 2.2. The Velocity-Independent Ratio

For a monostatic CW radar, the Doppler shift from a target with horizontal velocity *v* at incidence angle θ is:

(4) $$f_{d}=\frac{2vf_{0}}{c}\cos\left(\theta \right),$$

where $f_{0}$ is the carrier frequency and *c* the speed of light. Figure 3 visualizes the two contributions as *v* $\cos(\theta_{\mathrm{roof}})$ and *v* $\cos(\theta_{\mathrm{wheels}})$.

To make the velocity cancellation explicit:

$$\alpha =\frac{\left(\frac{2vf_{0}}{c}\right)\cos\left(\theta_{\mathrm{roof}}\right)}{\left(\frac{2vf_{0}}{c}\right)\cos\left(\theta_{\mathrm{wheels}}\right)}=\frac{\cos(\theta_{\mathrm{roof}})}{\cos(\theta_{\mathrm{wheels}})}.$$

Applying this relationship to each scattering center and taking their ratio:

(5) $$\alpha =\frac{f_{\mathrm{roof}}}{f_{\mathrm{wheels}}}=\frac{\cos(\theta_{\mathrm{roof}})}{\cos(\theta_{\mathrm{wheels}})}=\sqrt{\frac{R^{2}+h^{2}}{R^{2}+{\left(h-h_{0}\right)}^{2}}}.$$

The velocity *v* cancels algebraically, leaving α dependent only on geometry—the theoretical foundation of the proposed technique.

### 2.3. Height Calculation

Squaring Equation (5) and solving for $h_{0}$:

(6) $${\left(h-h_{0}\right)}^{2}=\frac{R^{2}+h^{2}-\alpha^{2}R^{2}}{\alpha^{2}}.$$

Taking the positive square root (valid for $h>h_{0}$, i.e., α≥1):

(7) $$h_{0}=h-\frac{1}{\alpha }\sqrt{R^{2}+h^{2}-\alpha^{2}R^{2}}.$$

This closed-form expression requires accurate measurement of *R* and α.

### 2.4. Simplified Model for Design Insight

For $R\gg h,h_{0}$, using the binomial approximation $\sqrt{R^{2}+x^{2}}\approx R\left(1+x^{2}/2R^{2}\right)$:

(8) $$\cos\left(\theta_{\mathrm{roof}}\right)\approx 1-\frac{{\left(h-h_{0}\right)}^{2}}{2R^{2}},\;\cos\left(\theta_{\mathrm{wheels}}\right)\approx 1-\frac{h^{2}}{2R^{2}}.$$

The Doppler ratio becomes:

(9) $$\alpha \approx 1+\frac{2hh_{0}-h_{0}^{2}}{2R^{2}},$$

which for $h_{0}\ll h$ simplifies to:

(10) $$\alpha -1\approx \frac{hh_{0}}{R^{2}}.$$

This relationship shows that α−1 is proportional to $h_{0}$ and inversely proportional to $R^{2}$, providing essential design intuition. All quantitative results in this paper use the exact expression (7).

### 2.5. Distance Measurement in Laboratory and Deployment

The accurate determination of horizontal distance *R* is essential for the height calculation in Equation (7). The methods employed differ between the laboratory validation and operational deployment scenarios.

#### 2.5.1. Laboratory Distance Measurement

In the scaled laboratory testbed, distance *R* was determined using two complementary methods:

1. **Photogate trigger:** An infrared photogate positioned at a known distance from the radar provides a precise trigger instant when the cart passes. This establishes a reference time $t_{\mathrm{trigger}}$ at known position $R_{\mathrm{trigger}}$.
2. **Optical video tracking:** A high-speed camera captures the cart motion throughout the experiment. Using the open-source Tracker video analysis software [26], the cart position is tracked frame-by-frame, yielding R(t) with typical accuracy of 1–2 mm after geometric calibration.

The combination of photogate triggering and video tracking provides accurate ground-truth distance data for validating the Doppler ratio model. The optical tracking system is used solely for validation purposes in the laboratory and is not required for operational deployment.

#### 2.5.2. Deployment Distance Measurement

For operational deployment on actual bridges, several practical approaches can provide the required distance measurement:

**FMCW Radar Ranging:** The same 94 GHz radar system can be reconfigured to operate in frequency-modulated continuous-wave (FMCW) mode to directly measure range. In FMCW mode, the transmitted frequency is swept linearly over a bandwidth *B*, and the range to a target is determined from the beat frequency:

(11) $$R=\frac{c\;\cdot \;f_{b}\;\cdot \;T_{\mathrm{sweep}}}{2B},$$

where $f_{b}$ is the measured beat frequency, $T_{\mathrm{sweep}}$ is the sweep period, and *B* is the chirp bandwidth. A dual-mode radar that alternates between CW Doppler mode (for height measurement) and FMCW mode (for ranging) can provide both α and *R* independently. For typical height-estimation zones with *R*∼20–50 m, a moderate bandwidth of 200 MHz provides range resolution of approximately 75 cm, suitable for initial target detection and approximate range gating. However, this dual-mode approach introduces practical challenges, including waveform scheduling, shared-aperture calibration, and potential mutual interference between ranging and Doppler-processing chains. In addition, a 75 cm range resolution may be insufficient for direct centimeter-level height estimation unless supported by averaging, calibration, or wider bandwidth.

**Inductive Loop Triggers:** Standard inductive loop sensors embedded in the roadway can provide precise vehicle detection at known positions. When a vehicle passes over a loop at surveyed distance $R_{\mathrm{loop}}$ from the radar, the loop trigger synchronized with the radar acquisition enables deterministic range assignment:

(12) $$R\left(t\right)=R_{\mathrm{loop}}-v\;\cdot \;\left(t-t_{\mathrm{trigger}}\right),\;t\geq t_{\mathrm{trigger}},$$

where $t_{\mathrm{trigger}}$ is the time when the loop was activated and the minus sign reflects an approaching vehicle (decreasing *R*). This approach leverages existing traffic infrastructure and provides position accuracy typically on the order of centimeters [8].

**Optical or Laser Curtain Triggers:** A low-cost infrared or laser beam positioned at a known distance $R_{\mathrm{trigger}}$ upstream of the measurement zone can provide event-based triggering. Upon beam interruption, the system initiates Doppler measurement at a precisely known geometry.

**Camera-Based Tracking:** For installations where cameras are already deployed for traffic monitoring, computer vision algorithms can estimate vehicle position, providing an additional source of range information.

These auxiliary methods (inductive loops, optical triggers, and camera cues) are optional deployment aids rather than methodological requirements. The core Doppler-ratio height-estimation principle remains radar-based and compatible with standalone operation.

#### 2.5.3. Recommended Implementation Strategy

For operational deployment, we consider three implementation architectures:

1. **Standalone radar architecture:** A dual-mode radar (CW Doppler + FMCW) can operate without external sensors. This preserves infrastructure independence but increases radar hardware and processing complexity.
2. **Infrastructure-assisted architecture (optional):** Fixed-geometry triggers (inductive loops/optical beams) provide deterministic range references, after which Doppler processing is performed in a short window (e.g., <50 ms at highway speeds), keeping propagation error low.
3. **Hybrid strategy:** Use infrastructure triggering for deterministic event timing and FMCW for coarse range tracking and multi-target disambiguation between trigger events.

These options represent a trade-off between deployment complexity, infrastructure requirements, and achievable range certainty; therefore, range-acquisition strategy should be selected per site constraints rather than treated as a fixed requirement of the Doppler-ratio method itself.

### 2.6. Frequency Separation and Radar Placement Considerations

To maximize measurement sensitivity and robustness, it is desirable to maximize the frequency separation:

(13) $$\Delta f_{d}=|f_{\mathrm{roof}}-f_{\mathrm{wheels}}|=\frac{2vf_{0}}{c}|\cos\left(\theta_{\mathrm{roof}}\right)-\cos\left(\theta_{\mathrm{wheels}}\right)|.$$

For fixed vehicle parameters $\left(v,h_{0}\right)$ and horizontal distance *R*, the radar height *h* influences $\Delta f_{d}$. In practical bridge installations, the radar mounting height is constrained by the physical infrastructure—typically limited to heights near or slightly above the bridge clearance itself.

For realistic deployment scenarios with R≈15–30 m and vehicle height $h_{0}\approx 4$–5 m, mounting the radar at heights of 5–8 m (i.e., slightly above the clearance threshold) provides adequate angular separation between the roof and wheel reflection paths. At these geometries, the frequency separation $\Delta f_{d}$ remains well above the spectral resolution achievable with short integration times, ensuring robust peak detection. The key practical requirement is that the radar must be positioned high enough to illuminate both the roof and wheel-level scatterers simultaneously, while maintaining sufficient angular difference to produce resolvable Doppler peaks.

### 2.7. Error Analysis and Integration Time

The height estimate in Equation (7) depends on the measured Doppler ratio $\alpha =f_{\mathrm{roof}}/f_{\mathrm{wheels}}$, which is derived from two spectral peaks in the short-time Fourier transform. The uncertainty in the estimated height $h_{0}$ due to uncertainties in the measured parameters can be analyzed using error propagation.

To first order, the uncertainty in $h_{0}$ due to an uncertainty δα in the Doppler ratio can be expressed as:

(14) $$\delta h_{0}\approx \left|\frac{\partial h_{0}}{\partial \alpha }\right|\delta \alpha .$$

Differentiating Equation (7) with respect to α using the chain rule, let $A=R^{2}+h^{2}-\alpha^{2}R^{2}$ for notational convenience. Then:

(15) $$h_{0}=h-\alpha^{-1}\sqrt{A}.$$

Computing the derivative and substituting $\partial A/\partial \alpha =-2\alpha R^{2}$:

(16) $$\frac{\partial h_{0}}{\partial \alpha }=\frac{R^{2}+h^{2}}{\alpha^{2}\sqrt{R^{2}+h^{2}-\alpha^{2}R^{2}}}.$$

This expression quantifies the sensitivity of the height estimate to errors in the Doppler ratio for a given geometry (h,R). The sensitivity increases as α approaches unity (i.e., when $h_{0}\to h$, making the two reflection paths nearly identical) and as the geometry approaches grazing incidence (large *R* for given *h*).

The dominant contribution to δα stems from the frequency resolution and peak-picking accuracy in the Doppler spectrum. For a rectangular time window of duration $T_{\mathrm{int}}$, the frequency resolution is approximately:

(17) $$\Delta f\approx \frac{1}{T_{\mathrm{int}}}.$$

Assuming that both Doppler peaks are estimated with an uncertainty on the order of the spectral resolution, and that the errors are uncorrelated:

(18) $$\delta \alpha \approx \Delta f\sqrt{\frac{1}{f_{\mathrm{roof}}^{2}}+\frac{1}{f_{\mathrm{wheels}}^{2}}}.$$

Sensitivity to range uncertainty can be written explicitly from Equation (7) as:

(19) $$\frac{\partial h_{0}}{\partial R}=\frac{R(\alpha^{2}-1)}{\alpha \sqrt{R^{2}+h^{2}-\alpha^{2}R^{2}}}.$$

To model systematic angular perturbations, let $\delta_{\theta }$ denote a common offset in both incidence angles. This offset represents mechanisms such as road-grade variations, structural deformation of the mounting bracket, or misalignment between the assumed horizontal reference and the actual velocity direction. In a CW Doppler system, the measured frequency depends on the geometric angle between the velocity vector and the radar–target line of sight; any mechanism that perturbs this angle by a common offset $\delta_{\theta }$ produces the bias modeled below. The perturbed ratio is:

(20) $$\alpha \left(\delta_{\theta }\right)=\frac{\cos(\theta_{\mathrm{roof}}+\delta_{\theta })}{\cos(\theta_{\mathrm{wheels}}+\delta_{\theta })},$$

and first-order expansion around $\delta_{\theta }=0$ gives:

(21) $${\left.\frac{\partial \alpha }{\partial \delta_{\theta }}\right|}_{\delta_{\theta }=0}=\alpha \left(\tan\theta_{\mathrm{wheels}}-\tan\theta_{\mathrm{roof}}\right).$$

Hencethe corresponding height perturbation is approximated by:

(22) $$\delta h_{0,\mathrm{tilt}}\approx \left|\frac{\partial h_{0}}{\partial \alpha }\right|\alpha \left|\tan\theta_{\mathrm{wheels}}-\tan\theta_{\mathrm{roof}}\right|\left|\delta_{\theta }\right|.$$

In Equations (20)–(22), $\delta_{\theta }$ is expressed in radians.

A compact systematic error budget can therefore be expressed as:

(23) $$\sigma_{h_{0}}^{2}\approx {\left(\frac{\partial h_{0}}{\partial \alpha }\sigma_{\alpha }\right)}^{2}+{\left(\frac{\partial h_{0}}{\partial R}\sigma_{R}\right)}^{2}+{\left(\frac{\partial h_{0}}{\partial \delta_{\theta }}\sigma_{\delta_{\theta }}\right)}^{2}+\sigma_{\mathrm{dyn}}^{2}+\sigma_{\mathrm{clutter}}^{2},$$

where $\sigma_{\mathrm{dyn}}$ captures vehicle pitch/roll and suspension dynamics, and $\sigma_{\mathrm{clutter}}$ captures peak bias from multipath/additional scatterers. Laboratory accuracy should therefore not be interpreted as guaranteed field accuracy; specifically, $\sigma_{\mathrm{dyn}}$ and $\sigma_{\mathrm{clutter}}$ require dedicated quantification in full-scale field campaigns.

Equations (16)–(23) highlight two key design considerations. First, the height estimate becomes more sensitive as the geometry approaches a grazing configuration, emphasizing the importance of radar standoff/range certainty and installation calibration. Second, longer integration times improve the frequency resolution (reducing Δf and hence δα), but at the cost of increased geometric variation during the window.

The strong dependence of $h_{0}$ on the horizontal distance *R* in Equation (7) further motivates the use of accurate distance measurement methods; this effect is quantified in Section 5.

## 3. System Design and Experimental Setup

### 3.1. Experimental Objectives

The laboratory experiments were designed with three main objectives: (1) to verify the Doppler ratio model by confirming that the radar-derived $\alpha =f_{\mathrm{roof}}/f_{\mathrm{wheels}}$ agrees with the angle-based ratio $\cos\left(\theta_{\mathrm{roof}}\right)/\cos\left(\theta_{\mathrm{wheels}}\right)$ derived from optical geometry; (2) to identify optimal measurement instants for each experimental run at which the radar-derived and geometry-derived values of α converge with high confidence; and (3) to quantify height estimation accuracy by applying the closed-form height formula in Equation (7) and comparing the estimated height to the true measured mast height.

### 3.2. Scaled Testbed Configuration

**Figure 4:**
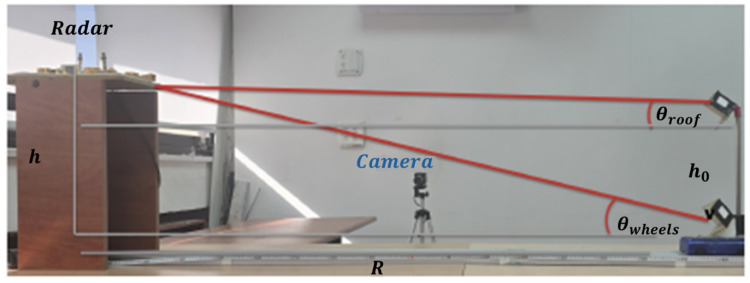
Experimental setup featuring the 94 GHz radar (left) and a scaled vehicle model on a linear track (right). The model includes an adjustable mast equipped with corner reflectors to simulate distinct scattering points. The overlaid annotations denote the geometric parameters used for height estimation, where $\theta_{\mathrm{roof}}$ and $\theta_{\mathrm{wheels}}$ represent the elevation angles to the upper and lower scatterers, respectively.

To validate the theoretical model under controlled conditions, a laboratory testbed was constructed as shown in Figure 4. The system consists of a motorized cart on a linear rail track approximately 3 m long to simulate a vehicle moving at constant velocity, an adjustable vertical mast mounted on the cart representing the truck height with the mast height varied across test cases from 20 cm to 46 cm, a 94 GHz continuous-wave Doppler radar module (Ancortek SDR-KIT 94V2) mounted on a fixed support structure above the track with the radar height adjusted between 38.5 cm and 64 cm depending on the test case, and a DC motor with encoder feedback maintaining the cart velocity at approximately 1.5 m/s.

The horizontal distance *R* between the cart’s initial position and the radar’s vertical projection was adjusted to emulate realistic bridge installation geometry when scaled to full size. The radar antenna was slightly tilted (approximately 5°) towards the direction of motion to ensure simultaneous illumination of both the roof-level and wheel-level regions throughout the measurement interval.

### 3.3. Corner Reflector Design and Placement

For full-scale vehicles (trucks, buses), the natural radar cross section (RCS) of the metallic surfaces—particularly the roof and wheel assemblies—is sufficient to produce strong, detectable returns at 94 GHz without any vehicle-side modifications. Typical commercial vehicle RCS values at W-band range from 10 m^2^ to 100 m^2^ (10–20 dBsm) [27], ensuring robust detection at operational distances of 20–50 m. The scattering characteristics of the vehicle’s features can be modeled as dihedral-like corner reflectors, providing stable phase centers for Doppler estimation [28]. In the laboratory setup, this behavior was emulated using small trihedral reflectors to ensure well-defined scattering centers for model validation. In practical deployment, the central challenge is often not signal strength per se, but stable and repeatable identification of the specific roof-level and wheel-level scattering components within a dense return cloud (e.g., grill, mirrors, cabin edges, undercarriage, and cargo geometry).

A small trihedral corner reflector with edge length of approximately 4 cm was mounted on top of the adjustable mast as the roof reflector. The reflector was fabricated using 3D printing (PLA plastic) and coated with conductive copper paint to provide a metallic surface. Following standard corner-reflector design practice at millimeter-wave frequencies [27,28], the edge length was chosen such that the far-field condition $r>2D^{2}/\lambda$ is satisfied at the operating distance, and the main lobe of the reflector’s radiation pattern (approximately ±20° from boresight) encompasses the expected angular variation during approach.

A second, smaller corner reflector (edge length ≈3 cm) was placed near the wheel-level region of the cart to ensure a strong and stable return from the lower reflection point. This was necessary because the rail and laboratory floor have low natural reflectivity at 94 GHz. The dual-reflector configuration yields two well-separated and persistent reflection points that can be tracked both in the radar spectra and in the camera images.

The theoretical RCS of a trihedral corner reflector is given by [27]:

(24) $$\sigma =\frac{4\pi a^{4}}{3\lambda^{2}},$$

where *a* is the edge length. For a=4 cm and λ=3.2 mm, this yields σ≈1.0 m^2^ (approximately 0 dBsm), which provides adequate signal-to-noise ratio for robust detection at the experimental distances (0.8–1.3 m). It should be emphasized that these corner reflectors were necessary only for the scaled laboratory demonstration; full-scale operational deployment would rely on the vehicle’s inherent radar reflectivity without requiring any vehicle-side modifications.

### 3.4. Ground Truth Validation with Tracker

This optical subsystem is included only to provide independent ground truth for model validation; the proposed Doppler-ratio height estimation does not rely on video tracking in deployment. An independent optical validation system was deployed to generate precise ground-truth data for position R(t), velocity v(t), and the geometric angles. The system consists of a high-speed global shutter camera (ELP 210fps Global Shutter USB Camera with OV9281 sensor, 720P resolution) positioned at an offset of approximately 1.2 m from the rail and at height 25 cm providing an oblique view of the cart motion, video recording at frame rates up to 210 fps to capture smooth trajectories, and the open-source Tracker video analysis software [26] for automated tracking of marked points on the cart.

The tracker provides sub-pixel localization of user-defined feature points, yielding time-stamped position coordinates with a typical accuracy of 1–2 mm at the camera distances used. From the tracked positions, the angles $\theta_{\mathrm{roof}}\left(t\right)$ and $\theta_{\mathrm{wheels}}\left(t\right)$ are computed, and hence the geometrically derived ratio:

(25) $$\alpha_{\mathrm{angles}}\left(t\right)=\frac{\cos\theta_{\mathrm{roof}}\left(t\right)}{\cos\theta_{\mathrm{wheels}}\left(t\right)}.$$

**Figure 5:**
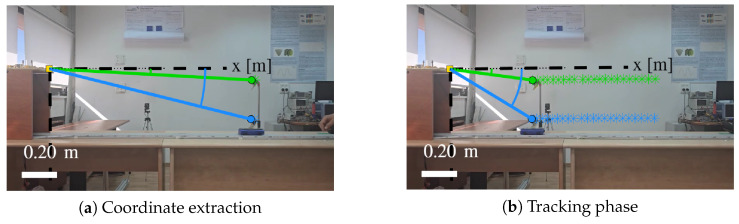
Tracker validation system used for ground-truth estimation (example shown for $h_{0}=0.20$ m). (**a**) Initial coordinate extraction phase at the beginning of the experiment. (**b**) Tracking phase, where the optical tracker continuously follows the roof and wheel corner reflectors to validate geometry and velocity.

**Figure 6:**
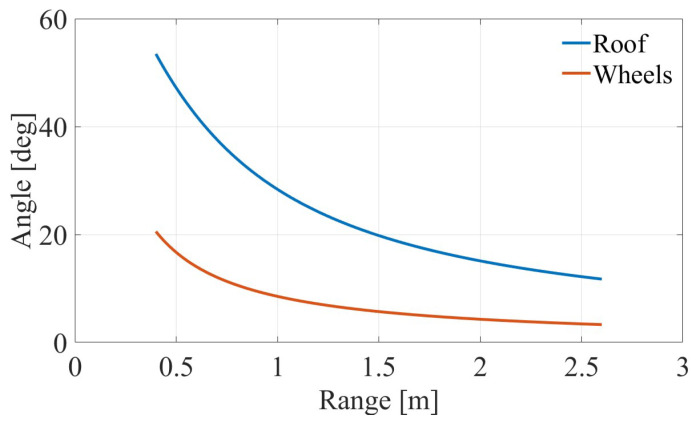
Incidence angle versus range extracted from video processing, showing the angular variation of the roof and wheel reflectors during the approach trajectory.

Representative frames from the Tracker pipeline are shown in Figure 5. The resulting angular variation for both reflectors during the approach trajectory is illustrated in Figure 6, which plots the incidence angle as a function of the horizontal range *R*. This optical ground truth provides an independent reference against which the radar-derived measurements can be validated.

### 3.5. Timing Synchronization

Precise temporal alignment between the radar and camera data streams is essential for meaningful comparison. Synchronization was achieved using a photogate (infrared LED–photodiode pair) placed near the beginning of the rail track. When the cart crosses the gate, the photogate triggers the radar data acquisition system via a TTL pulse starting the radar recording, simultaneously the same trigger activates a visible LED mounted in the camera’s field of view, and the LED flash is clearly visible in the video recording and serves as a time marker (t=0).

In roadway deployment, the measurement window can be triggered using standard infrastructure sensors (e.g., photogates or inductive loops) or by radar-internal detection logic.

Post-processing uses this common time reference to align both radar and video time axes. The overall timing accuracy is limited by the video frame rate (210 fps ⇒ 4.8 ms per frame) and the radar sampling rate (typically 10 kHz baseband sampling). For the cart velocities used (∼1.5 m/s), a 5 ms timing uncertainty corresponds to a spatial uncertainty of less than 1 cm, which is acceptable given the overall experimental accuracy targets.

## 4. Signal Processing: Core Principle and Integration Time

### 4.1. Core Measurement Principle

The 94 GHz radar operates in continuous-wave (CW) mode with homodyne (direct downconversion) detection. The transmitted signal can be represented as:

(26) $$s_{\mathrm{TX}}\left(t\right)=A_{0}\cos\left(2\pi f_{0}t\right),$$

where $f_{0}=94$ GHz is the carrier frequency.

The signal reflected from a moving target at distance r(t) with radial velocity $v_{r}$ undergoes a Doppler frequency shift. For a monostatic radar, the total phase shift for two-way propagation is:

(27) $$\phi \left(t\right)=\frac{4\pi }{\lambda }r\left(t\right)=\frac{4\pi f_{0}}{c}r\left(t\right).$$

The received signal from a single scatterer is:

(28) $$s_{\mathrm{RX}}\left(t\right)=A_{r}\cos\left(2\pi f_{0}t+\frac{4\pi f_{0}}{c}r\left(t\right)\right).$$

After mixing with the local oscillator and low-pass filtering, the baseband signal contains the Doppler term:

(29) $$s_{\mathrm{BB}}\left(t\right)\propto \cos\left(\frac{4\pi f_{0}}{c}r\left(t\right)\right).$$

For a target moving with constant radial velocity $v_{r}$, $r\left(t\right)=r_{0}-v_{r}t$, and the baseband signal becomes:

(30) $$s_{\mathrm{BB}}\left(t\right)\propto \cos\left(2\pi f_{d}t+\phi_{0}\right),$$

where $f_{d}=2v_{r}f_{0}/c$ is the Doppler frequency.

In the present application, the vehicle presents two dominant scatterers (roof and wheels), yielding a composite baseband signal:

(31) $$s_{\mathrm{BB}}\left(t\right)=A_{1}\cos\left(2\pi f_{\mathrm{roof}}t+\phi_{1}\right)+A_{2}\cos\left(2\pi f_{\mathrm{wheels}}t+\phi_{2}\right).$$

This superposition produces a characteristic beat pattern in the time domain: a fast oscillation at the frequency of the dominant reflector, amplitude-modulated by a slower envelope at the difference frequency $\Delta f=|f_{\mathrm{roof}}-f_{\mathrm{wheels}}|$.

**Figure 7:**
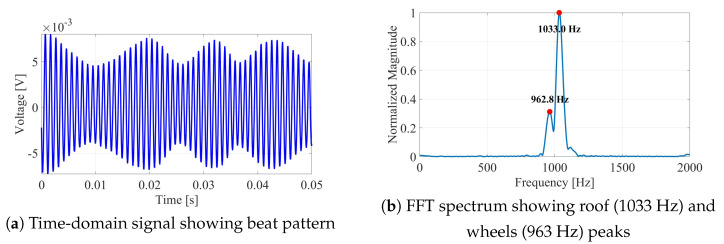
Core measurement principle: (**a**) raw radar signal showing the beat pattern characteristic of two closely spaced Doppler frequencies; (**b**) FFT spectrum for a 20 cm target with α ratio convergence analysis.

Figure 7 illustrates the core processing pipeline for a representative test case (mast height 20 cm).

The processing pipeline consists of six sequential stages. First, the raw baseband signal (Figure 7a) is sampled at 10 kHz and recorded for the duration of the cart’s transit through the radar beam, typically spanning 2–3 s. Following data acquisition, a short-time Fourier transform (STFT) is applied to extract Doppler frequencies from time windows of duration $T_{\mathrm{int}}$. Figure 7b presents a representative spectrum revealing two distinct peaks corresponding to the roof and wheels reflectors. Peak extraction then uses physically constrained selection: candidate peaks are searched in expected positive-Doppler bands, a minimum frequency separation is enforced, and temporal persistence across consecutive windows is required before assigning roof/wheel labels. In the controlled laboratory data this procedure reduces to selecting two dominant peaks, but the formulation is designed to extend to cluttered scenes. The radar-derived $\alpha_{\mathrm{radar}}=f_{\mathrm{roof}}/f_{\mathrm{wheels}}$ is then compared with the geometry-derived $\alpha_{\mathrm{angles}}\left(t\right)$ obtained from the Tracker data. These two independent estimates converge at a specific time interval, identifying the optimal measurement instant $t^{★}$. Finally, at time $t^{★}$, the horizontal distance $R(t^{★})$ is extracted from the Tracker trajectory data, enabling height calculation via Equation (7).

### 4.2. Integration Time and Frequency Resolution

The Doppler frequencies $f_{\mathrm{roof}}$ and $f_{\mathrm{wheels}}$ are extracted using a discrete Fourier transform over a time window of duration $T_{\mathrm{int}}$. For a rectangular window, the frequency resolution (bin spacing) is:

(32) $$\Delta f=\frac{1}{T_{\mathrm{int}}}.$$

The choice of $T_{\mathrm{int}}$ involves a fundamental trade-off. Short integration times ensure the vehicle moves only a short distance during the window, so the geometry $\left(R,\theta_{\mathrm{roof}},\theta_{\mathrm{wheels}}\right)$ remains approximately constant, but poor frequency resolution may prevent the two peaks from being resolved. Long integration times provide high frequency resolution but introduce spectral broadening due to changing geometry.

The optimal $T_{\mathrm{int}}$ must balance these competing effects and depends on the vehicle height $h_{0}$ (which determines the frequency separation), the vehicle velocity *v* (which determines geometric change rate), and the horizontal distance *R* (which affects angular rates).

### 4.3. Integration Time Analysis in the Scaled Experiment

In the scaled laboratory setup, the cart velocity is $v_{\mathrm{lab}}\approx 1.5$ m/s. The impact of integration time is illustrated by comparing two representative cases.

For a tall vehicle (40 cm mast) with h=60.5 cm, $h_{0}=40$ cm, R≈0.83 m, from Equation (5), α≈1.200. At v=1.5 m/s and 94 GHz, the frequency separation is $\Delta f_{d}\approx 180$ Hz. A $T_{\mathrm{int}}=25$ ms window provides Δf=40 Hz resolution, sufficient to resolve the peaks. During 25 ms, the cart moves 3.75 cm, corresponding to an angular change of approximately 2.7°—small enough not to significantly broaden the peaks.

For a short vehicle (20 cm mast) with h=42.0 cm, $h_{0}=20$ cm, R≈0.84 m, from Equation (5), α≈1.082. The frequency separation is $\Delta f_{d}\approx 80$ Hz. A longer window ($T_{\mathrm{int}}=50$ ms, Δf=20 Hz) is necessary to achieve clear peak separation, though during 50 ms, the cart moves 7.5 cm, introducing more geometric variation.

**Figure 8:**
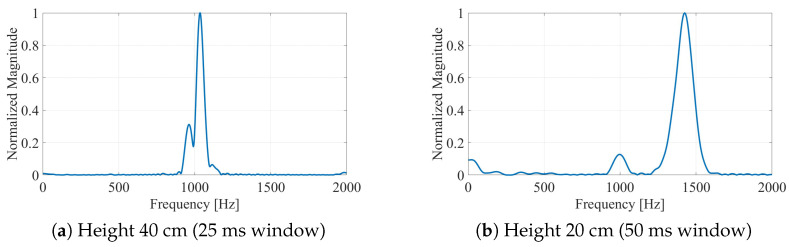
Integration-time effect: (**a**) for 40 cm height, a 25 ms window provides sufficient resolution (∼40 Hz) to separate the large Δf; (**b**) for 20 cm height, a 50 ms window is required to achieve 20 Hz resolution.

Figure 8 demonstrates this contrast experimentally.

**Figure 9:**
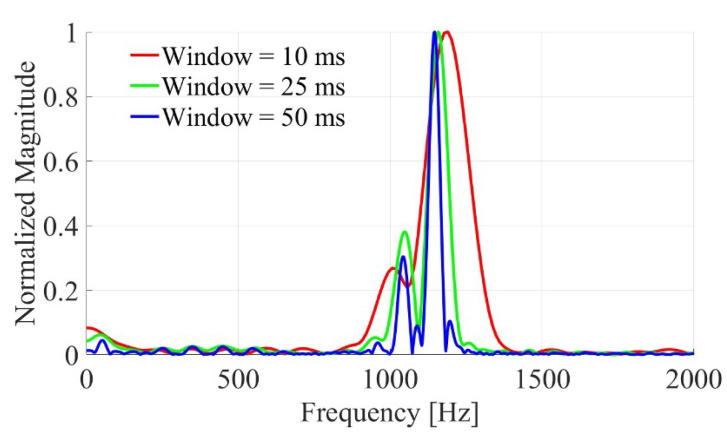
Demonstration of integration time effect on spectral resolution: comparison of FFT results for different window lengths (10 ms, 25 ms, and 50 ms) showing the trade-off between frequency resolution and spectral broadening.

Figure 9 further illustrates the integration-time trade-off by comparing FFT spectra obtained with different window lengths (10 ms, 25 ms, and 50 ms), highlighting the balance between frequency resolution and spectral broadening.

These observations confirm the theoretical trade-off and inform the selection of integration time for each test case. In practice, an adaptive algorithm could initially use a short window to coarsely estimate α and the vehicle height, then select an appropriate $T_{\mathrm{int}}$ based on the expected frequency separation.

### 4.4. Peak Association Under Multiple Roof Scatterers

To further evaluate robustness under multi-scattering-center conditions, an additional experiment was conducted by introducing multiple reflective elements above the roof region. For a scatterer at vertical coordinate *z* (measured from road level), the Doppler component is:

(33) $$f_{d}\left(z\right)=\frac{2vf_{0}}{c}\frac{R}{\sqrt{R^{2}+{\left(h-z\right)}^{2}}}.$$

For 0≤z<h, Equation (33) is monotonic, increasing with *z*, meaning that the physically higher scatterer yields a higher Doppler frequency magnitude. In addition to the wheel-level and roof-level corner reflectors, a thin metallic rod and a small flat metallic plate were placed successively above the roof-level reflector, producing four distinct peaks in the FFT spectrum.

**Figure 10:**
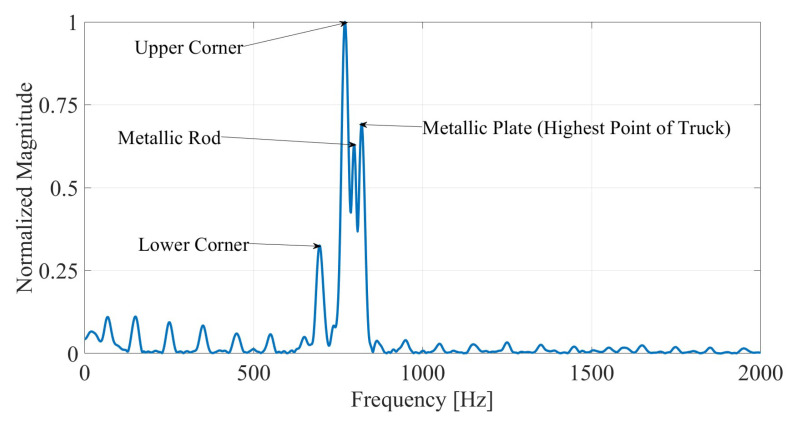
Doppler FFT spectrum under multi-scattering-center conditions. Four Doppler peaks correspond to the lower corner reflector, upper corner reflector, thin metallic rod, and upper metallic plate. The physically highest element produces the highest Doppler frequency, although not necessarily the strongest peak in amplitude.

Figure 10 shows the corresponding spectrum. The peaks appear at increasing frequencies corresponding to increasing physical height. Notably, the highest Doppler frequency is associated with the uppermost scatterer (metallic plate), although its reflected amplitude is not necessarily the largest. The thin metallic rod produces a weaker peak due to its limited radar cross section, yet its Doppler frequency correctly reflects its relative height.

These results show that the proposed method identifies the uppermost structure using Doppler frequency ordering rather than signal amplitude alone. This criterion does not eliminate all field challenges (e.g., strong clutter, overlapping vehicles, wet-road scattering changes), but it provides a physically grounded basis for peak association when multiple roof-related scatterers exist.

Frequency ordering addresses association only when the relevant peaks are detectable. If the upper-structure peak falls below the detection threshold, the required dual-peak condition is not met, precluding height estimation in the current formulation; operationally, this is treated as a no-decision state. A more subtle failure mode is underestimation when an intermediate-height scatterer is stronger than the true roof return and peak selection is driven by amplitude. For bridge-protection applications, this motivates assigning the roof candidate as the highest-frequency persistent peak above the SNR threshold, not the highest-amplitude peak. For the primary target class considered here (flat-roofed metallic containers and buses), the roof return is typically strong at the W-band; however, validation under irregular cargo/load geometries remains necessary.

## 5. Experimental Results

### 5.1. Sensitivity of Height Estimate to Distance

**Figure 11:**
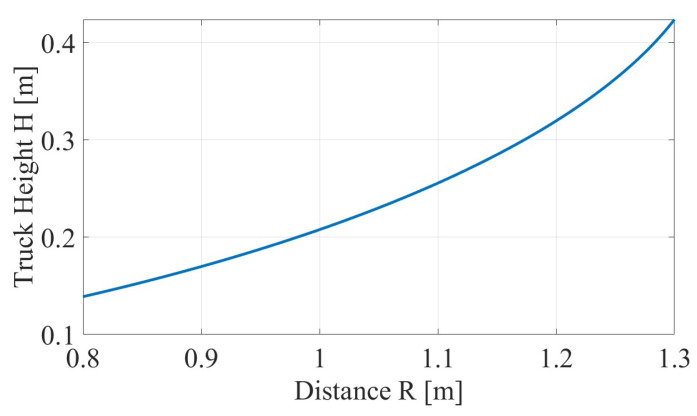
Height calculation sensitivity: dependence of estimated $h_{0}$ on distance *R* for fixed h=54 cm and α=1.086. The steep slope at larger distances highlights the need for accurate determination of *R*.

As evident from Equation (7), the estimated height $h_{0}$ is a nonlinear function of the horizontal distance *R*. Figure 11 illustrates this dependence for representative experimental parameters.

The plot shows that even small errors in *R* can propagate to noticeable deviations in the estimated height, particularly when the geometry approaches grazing incidence. For example, at R=1.2 m with α=1.086, a 5 cm error in *R* translates to approximately 1 cm error in $h_{0}$.

In this study, the optical system is used to accurately determine *R* at the selected measurement instant for validation. In full-scale implementations, *R* can be obtained via FMCW ranging, auxiliary ranging sensors, or calibrated installation geometry.

### 5.2. Sensitivity to Systematic Angular Perturbations

Using Equation (22), we evaluated the first-order effect of a common angular perturbation for representative geometries from Table 1. Table 2 summarizes the predicted height-bias magnitude for ±0.5° and ±1° perturbation.

**Figure 12:**
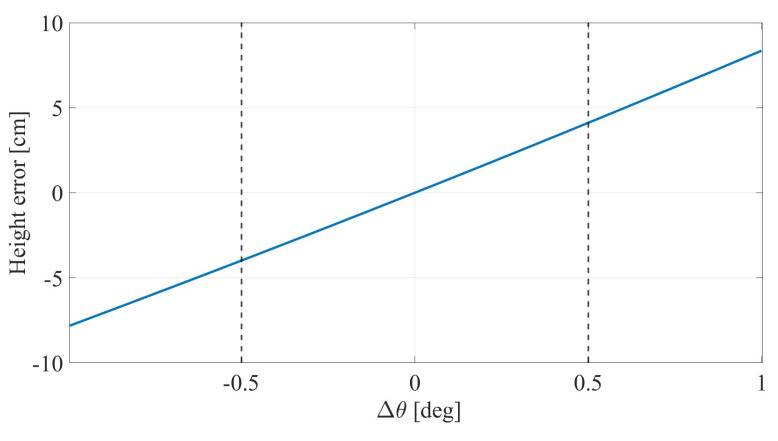
Estimated height bias versus systematic angular perturbation Δθ (e.g., road grade or reference-plane misalignment) for a representative geometry (Case 6 in Table 2), showing the near-linear dependence predicted by Equation (22). Dashed lines indicate the practical control interval around ±0.5°.

Figure 12 shows an illustrative angular-perturbation sensitivity curve for representative system parameters, highlighting the approximately linear dependence and the practical control interval around ±0.5°.

The analysis shows approximately linear growth of height bias with common angular perturbation, consistent with Equation (22). Since Equation (22) is a first-order model, the ±1° values should be interpreted as indicative bounds; exact nonlinear evaluation may introduce mild asymmetry. Practically, this motivates explicit installation calibration, site geometry surveying, and periodic alignment verification. The method remains viable under non-ideal mounting, but angular-perturbation uncertainty should be included in the deployment error budget.

### 5.3. Quantitative Results and Error Statistics

Extensive trials were conducted across six distinct test cases, spanning mast heights from 20 cm to 46 cm and various radar heights and distances. Table 1 summarizes the key results.

Several key observations emerge from these results. The system achieves good accuracy across all test cases, with an average absolute error of approximately 0.60 cm and average relative error of 1.65%. The radar-derived α values consistently fall within the range of geometrically derived values from Tracker, confirming the validity of the theoretical model.

The largest error occurs in Case 6 (46 cm height, −1.5 cm error, 3.26% relative). This case had the largest horizontal distance (R=1.26 m), where the angular difference between the two paths is smallest. At large *R*, the sensitivity to measurement noise increases, as predicted by the error analysis in Section 2. The best accuracy is achieved in Case 1 (20 cm height, 0.1 cm error, 0.50% relative), demonstrating that with appropriate integration time selection (50 ms in this case), even small vehicles can be accurately measured.

### 5.4. Worked Example: 35 cm Mast Height

To illustrate the complete processing chain, we detail the analysis for Case 4 (true mast height $h_{0}=35$ cm).

**Step 1: Spectral analysis.** From the FFT corresponding to a 25 ms integration window, the two Doppler peaks are identified:

(34) $$\begin{array}{cc} f_{\mathrm{roof}} & \approx 1089\;\mathrm{Hz}, \end{array}$$

(35) $$\begin{array}{cc} f_{\mathrm{wheels}} & \approx 1003\;\mathrm{Hz}. \end{array}$$

The radar-derived Doppler ratio is:

(36) $$\alpha_{\mathrm{radar}}=\frac{f_{\mathrm{roof}}}{f_{\mathrm{wheels}}}=\frac{1089}{1003}\approx 1.086.$$

**Step 2: Geometric validation.** The Tracker-based optical system provides $\alpha_{\mathrm{angles}}\left(t\right)\in \left[1.084,1.096\right]$ during the convergence interval around $t^{★}\approx 0.85$ s, with $\alpha_{\mathrm{radar}}=1.086$ lying within this range.

**Step 3: Distance determination.** From the Tracker trajectory data at $t=t^{★}$:

(37) $$R(t^{★})\approx 1.19\;m.$$

**Step 4: Height calculation.** With radar height h=54 cm, substituting into Equation (7):

(38) $$\begin{array}{cc} h_{0} & =0.54-\frac{1}{1.086}\sqrt{1.19^{2}+0.54^{2}-1.086^{2}\times 1.19^{2}}\\ \; & =0.54-\frac{1}{1.086}\sqrt{1.416+0.292-1.668}\\ \; & =0.54-\frac{1}{1.086}\sqrt{0.040}\approx 0.356\;m=35.6\;\mathrm{cm}. \end{array}$$

Rounding to the nearest 0.5 cm yields $h_{0}\approx 35.5$ cm.

**Figure 13:**
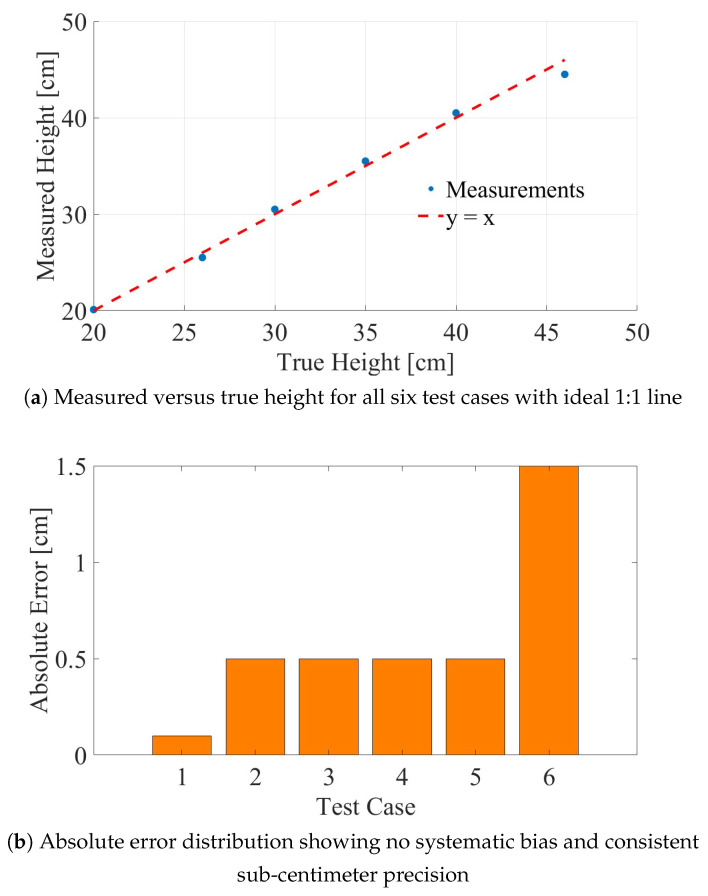
Measurement accuracy summary: (**a**) measured versus true height for all six test cases with ideal 1:1 line; (**b**) absolute error distribution showing no systematic bias and consistent sub-centimeter precision.

**Step 5: Error assessment.** The measured height differs from the true height of 35.0 cm by only 0.5 cm, corresponding to a relative error of 1.4%. This accuracy is further illustrated in Figure 13, which presents a comprehensive visualization of the measurement results across all experimental test cases.

### 5.5. Measurement Accuracy Visualization

Figure 13 presents a comprehensive visualization of the measurement accuracy achieved across all experimental test cases.

The upper panel displays the measured vehicle height plotted against the true height, overlaid with an ideal 1:1 correspondence line. The data points cluster tightly around this reference line, demonstrating good agreement. The lower panel presents the absolute measurement error for each test case, revealing no strong systematic bias across the height range. Five of the six cases exhibit absolute errors below 0.6 cm, with only Case 6 showing an elevated error of 1.5 cm—consistent with the increased horizontal distance employed in that geometry.

The small systematic errors observed (predominantly positive for smaller heights, negative for the largest height) may be attributed to residual uncertainties in corner reflector positioning, small variations in cart velocity (±5%), and Tracker calibration accuracy (±2 mm).

## 6. Scalability and Real-World Implementation

A critical question for any laboratory-scale proof-of-concept is whether the results translate to real-world operating conditions. The scaling analysis here includes both geometric and kinematic factors.

### 6.1. Geometric Scaling Summary

Table 3 summarizes representative laboratory-to-field mapping values used in this study. Unlike velocity, geometric deployment parameters are not required to scale linearly from the laboratory setup. In practice, height-estimation standoff and warning distance are design variables chosen per site constraints and safety policy.

The upper bound $R_{\mathrm{meas}}=50$ m is consistent with the design-distance analyses shown in Figure 1 and Figure 2. In deployment, this measurement zone is combined with upstream placement ($R_{\mathrm{warn}}$) to provide sufficient warning and braking distance before the clearance point.

### 6.2. Velocity Scaling and Doppler Shift

In the laboratory, the cart velocity was $v_{\mathrm{lab}}\approx 1.5$ m/s. A typical highway truck travels at $v_{\mathrm{real}}\approx 80$ km/h ≈22.2 m/s. The velocity scaling factor is:

(39) $$S_{v}=\frac{v_{\mathrm{real}}}{v_{\mathrm{lab}}}=\frac{22.2}{1.5}\approx 14.8.$$

Since the Doppler shift is directly proportional to velocity, all Doppler frequencies scale linearly:

(40) $$f_{\mathrm{Doppler},\mathrm{real}}=S_{v}\times f_{\mathrm{Doppler},\mathrm{lab}}.$$

For example, in the laboratory (Case 1, 20 cm height), the frequency separation was $\Delta f_{d}\approx 80$ Hz. On a real highway:

(41) $$\Delta f_{d}^{\mathrm{real}}\approx 14.8\times 80\approx 1.2\;\mathrm{kHz}.$$

### 6.3. Integration Time Implications

To complement the velocity-only scaling above, we evaluate a representative full-scale geometry over a practical height-estimation zone. For v=22.2 m/s, h=7 m, $h_{0}=4$ m, and $f_{0}=94$ GHz:

(42) $$\begin{array}{cc} \Delta f_{d}\left(R=20\;m\right) & \approx 0.64\;\mathrm{kHz}, \end{array}$$

(43) $$\begin{array}{cc} \Delta f_{d}\left(R=50\;m\right) & \approx 0.11\;\mathrm{kHz}, \end{array}$$

using Equation (13).

If at least 2–4 spectral bins are required between peaks, the target frequency resolution is approximately $\Delta f\approx \Delta f_{d}/2$ to $\Delta f_{d}/4$, which gives:

(44) $$T_{\mathrm{int}}^{\mathrm{real}}\approx \frac{1}{\Delta f}\approx 3–18\;\mathrm{ms},$$

for $R_{\mathrm{meas}}\approx 20$–50 m, depending on standoff range and desired detection margin.

The 2–4-bin design rule is intentionally practical rather than theoretical. A 1-bin separation is the Rayleigh limit for a rectangular window, but robust peak picking for α estimation requires margin against leakage and finite SNR. In practice, about 2 bins is the minimum in favorable conditions, while 3–4 bins provide more reliable separation when tapered windows (e.g., Hann) and moderate-SNR operation are considered.

### 6.4. Geometric Constancy at Highway Speeds

At 80 km/h (22.2 m/s), a vehicle travels:

(45) $$\Delta x=v\times T_{\mathrm{int}}=22.2\times 0.003\approx 0.067\;m=6.7\;\mathrm{cm}$$

during a 3 ms integration window (lower bound of Equation (44)). For an 18 ms window, Δx≈40 cm.

For a representative near-zone geometry with R≈20 m, a displacement of 6.7 cm corresponds to an angular change of:

(46) $$\Delta \theta \approx \frac{\Delta x}{R}\approx \frac{0.067}{20}\approx 0.0033\;\mathrm{rad}\approx 0.19{}^{\circ}.$$

At the upper integration bound (18 ms) with R≈50 m, Δθ≈0.40/50≈0.008rad≈0.46°.

This angular variation remains small compared to the laboratory case (where Δθ≈2.7° for 25 ms at R≈0.8 m). Thus, geometric smearing is expected to remain manageable in the targeted millisecond integration regime.

### 6.5. Favorable Scaling Trends

The scaling analysis reveals several favorable trends that suggest the laboratory results may translate to real-world conditions:

- Real-world vehicles produce Doppler frequencies approximately 15 times larger, enabling easier spectral separation.
- Required integration remains in the millisecond regime (approximately 3–18 ms for $R_{\mathrm{meas}}\approx 20$–50 m), allowing repeated updates as the vehicle approaches.
- Warning horizon can be extended independently of $R_{\mathrm{meas}}$ by upstream deployment ($R_{\mathrm{warn}}\approx 60$–120 m), corresponding to approximately 2.7–5.4 s at 80 km/h.
- Full-scale vehicles have larger radar cross sections than laboratory corner reflectors, potentially providing stronger signal returns.

However, real-world deployments will introduce additional challenges not present in the laboratory, including multipath reflections from the road surface, clutter from roadside objects, simultaneous returns from multiple vehicles, and varying environmental conditions. Field trials are essential to quantify these effects and validate system performance.

### 6.6. Practical Implementation Considerations

For deployment on an actual bridge, the following system design aspects should be addressed:

- A narrow-beam antenna (e.g., horn antenna with ∼5° beamwidth) should focus energy on the approaching lane and minimize clutter.
- With millisecond integration times, many independent measurements can be obtained as the vehicle approaches; statistical averaging or Kalman filtering can further improve accuracy.
- Standalone radar deployment is feasible with radar-only ranging (e.g., CW+FMCW). Integration with cameras or road sensors remains optional and may be used where such infrastructure already exists.
- A practical layout is to place the radar on a dedicated roadside pole upstream of the bridge, decoupling early-warning distance ($R_{\mathrm{warn}}$) from local height-estimation geometry ($R_{\mathrm{meas}}$).
- While 94 GHz offers good fog and rain penetration, heavy snow or ice accumulation on the antenna should be mitigated using radomes or heating elements.
- Threshold-based classification, temporal persistence tests, and multi-frame confirmation can reduce false alarms from birds, debris, or sensor noise.

## 7. Discussion and Future Work

### 7.1. Comparison with Existing Technologies

Table 4 summarizes technology trade-offs with emphasis on evidence level. The proposed method is reported with laboratory-validated accuracy only; field metrics and lifecycle cost remain to be quantified.

The proposed approach offers velocity independence and promising laboratory accuracy. Relative to FMCW-only methods, the core advantage is direct height inference from a Doppler ratio without explicit speed estimation. However, deployment-level claims require dedicated field validation.

### 7.2. Limitations and Challenges

Despite the promising results, several limitations should be acknowledged:

**Range dependency:** As demonstrated in Figure 11 and Equation (19), the height estimate is sensitive to errors in *R*. In real-world deployment, range uncertainty must be controlled by calibrated geometry, FMCW ranging, triggers, or hybrid methods.

**Systematic angular perturbation sensitivity:** As quantified in Table 2, a common angular perturbation (e.g., road grade, structural deformation, or reference-plane misalignment) can introduce non-negligible bias. Installation and maintenance procedures therefore require angular calibration and periodic alignment verification.

**Adverse weather and wet-road effects not experimentally verified:** The weather argument in this work is based on propagation physics and literature, not on dedicated fog/rain/wet-road experiments in the current setup. At 94 GHz, adverse-weather propagation impact can be expressed via the specific attenuation $\gamma_{w}$ (dB/km). For the stand-off ranges considered here (R≈15–50 m), the additional two-way loss is approximately $\Delta L_{w}\approx 2\gamma_{w}R/1000$ dB. Accordingly, for $\gamma_{w}=3$ dB/km (moderate rain), $\Delta L_{w}$ is about 0.09–0.30 dB; even for severe events with $\gamma_{w}$ on the order of 10–20 dB/km, $\Delta L_{w}$ is roughly 0.3–2 dB.

Wet-road effects mainly alter backscatter amplitude (detection margin) rather than the underlying Doppler geometry. Since α is formed from Doppler frequencies, wet surfaces do not directly change the ideal geometric mapping, but lower SNR and clutter can still bias practical peak estimation. At 94 GHz and near-grazing incidence, measurements report reduced wet-asphalt backscatter relative to dry surfaces due to water-film smoothing and increased specular behavior [29,30]. Although those datasets use automotive-like grazing angles, not the bridge-mounted geometry used here, the mechanism suggests that wheel-level SNR can degrade in some scenes. In the present setup, wheel-level returns are generally dominated by metallic wheel/undercarriage components, so the expected wet-road effect is primarily reduced detection margin; quantitative characterization versus vehicle class, rain intensity, and geometry remains future field work.

**Multi-lane and multi-target traffic:** The laboratory testbed considered a single target. Real roadways may include overlapping returns from several vehicles and roadside scatterers; beamforming/spatial filtering and target association are needed.

**Complex vehicle geometries and additional scatterers:** Real vehicles do not provide ideal point reflectors. The frequency-ordering criterion in Equation (33) helps, but robust association under clutter still requires further validation.

**Systematic error budget in field conditions:** Beyond α estimation error, pitch/roll dynamics, multipath, and finite beamwidth effects must be jointly quantified as in Equation (23).

### 7.3. Future Research Directions

Several avenues for future research are identified:

- Full-scale field trials on actual bridges with real traffic (subject to the required regulatory and safety approvals) to validate scaling predictions.
- Controlled environmental campaigns (fog, rain intensity, wet-road conditions) to quantify robustness limits.
- Real-time embedded systems (FPGA or DSP-based) for operational deployment.
- Extension to maritime applications for detecting over-height vessels approaching low bridges.
- Advanced signal processing techniques (MUSIC, ESPRIT, model-based tracking, and machine-learning-assisted peak association) for cluttered low-SNR scenarios.
- Multi-sensor fusion with complementary technologies (cameras, FMCW radar, lidar) using Bayesian or deep learning frameworks.

## 8. Conclusions

This study has presented an analysis of a 94 GHz millimeter-wave radar system for remote vehicle height measurement, with the objective of preventing collisions between over-height vehicles and low-clearance bridges or tunnels. The key contribution lies in the exploitation of the Doppler effect to eliminate explicit dependence on vehicle speed, thereby simplifying the measurement process and enhancing robustness.

A theoretical framework was developed, deriving the relationship between the Doppler frequency ratio $\alpha =f_{\mathrm{roof}}/f_{\mathrm{wheels}}$ and the geometric parameters. The closed-form height estimation formula in Equation (7) was validated experimentally and shown to be accurate across a range of vehicle heights and geometries. A simplified approximate model in Equation (9) was also introduced to provide design guidelines.

The trade-offs associated with FFT integration time were analyzed, highlighting the balance between frequency resolution and geometric constancy. Experimental results from a scaled laboratory testbed demonstrated that the proposed method achieves relative errors consistently below 3.3% and an average absolute error of approximately 0.60 cm across six representative test cases, and that the radar-derived Doppler ratio agrees well with the geometrically derived ratio from an independent optical tracking system.

A scaling analysis indicates that full-scale highway implementations should benefit from larger Doppler shifts (due to higher velocities), allowing millisecond integration windows in practical measurement zones (approximately 3–18 ms for $R_{\mathrm{meas}}\approx 20$–50 m, depending on geometry and detection margin). Warning distance can be extended independently by upstream sensor placement. However, real-world factors including multipath, clutter, adverse weather, and multi-vehicle scenarios require dedicated field validation before operational deployment.

The proposed system offers several advantages compared to existing technologies: the Doppler ratio method does not require prior knowledge of the vehicle speed; for full-scale vehicles, no vehicle-side equipment is required as the method relies on inherent radar reflectivity (corner reflectors were used in the scaled laboratory demonstration only to compensate for the reduced RCS of the miniature test platform); the system can be adapted to different bridge geometries and traffic scenarios; and the radar can be integrated with other sensors in a multi-layer sensing architecture. The expected weather resilience of 94 GHz radar is supported by known propagation characteristics, but quantitative weather-robustness metrics for this specific method remain future work.

With further development and validation through full-scale field trials, the proposed 94 GHz radar system has the potential to contribute to the safety and efficiency of transportation infrastructure.

## Figures and Tables

**Table 1:** Experimental results for six test cases in the scaled setup.

Case #	True $h_{0}$ (cm)	Radar *h* (cm)	α (Radar)	α Range (Tracker)	Dist *R* (m)	Meas $h_{0}$ (cm)	Abs Err (cm)	Rel Err (%)
1	20.0	42.0	1.082	1.075–1.095	0.84	20.1	0.1	0.50
2	26.0	38.5	1.050	1.045–1.053	1.125	25.5	−0.5	1.90
3	30.0	53.5	1.135	1.115–1.145	0.89	30.5	0.5	1.60
4	35.0	54.0	1.086	1.084–1.096	1.19	35.5	0.5	1.40
5	40.0	60.5	1.200	1.180–1.220	0.83	40.5	0.5	1.24
6	46.0	64.0	1.108	1.108–1.123	1.26	44.5	−1.5	3.26
Average absolute error:							0.60	
Average relative error:								1.65

**Table 2:** Predicted height bias from systematic angular perturbation ($\delta_{\theta }$).

Case	$h_{0}$ (cm)	*h* (cm)	*R* (m)	$|\delta h_{0}|$ for ±0.5°/±1° (cm)
1	20	42	0.84	0.71 / 1.42
4	35	54	1.19	1.96 / 3.92
6	46	64	1.26	2.87 / 5.73

**Table 3:** Representative laboratory-to-field mapping parameters (non-linear by design).

Parameter	Laboratory Value	Representative Field Value	Mapping Note
Vehicle roof height $h_{0}$	0.20–0.46 m	3.8–4.8 m	Vehicle class dependent
Radar height *h*	0.385–0.64 m	6–8 m	Chosen by infrastructure/clearance constraints
Height-estimation standoff $R_{\mathrm{meas}}$	0.8–1.3 m	20–50 m	Chosen for robust roof/wheel Doppler separation
Warning standoff to clearance point $R_{\mathrm{warn}}$	–	60–120 m	Chosen by reaction/braking policy
Vehicle speed *v*	1.5 m/s	15–25 m/s (54–90 km/h)	Dominant linear Doppler scaling term

**Table 4:** Comparison of over-height detection technologies with evidence context.

Technology	Weather Robustness	Velocity Independence	Cost Evidence	Performance Evidence
Infrared curtain	Limited in fog/rain and prone to nuisance interruptions	Yes	$25 k–$100 k/site reported for deployed systems [2,4]	Operationally used; false triggers reported in practice [2]
Laser scanner	High line-of-sight precision but weather/contamination sensitive	No	Site-specific, generally higher than simple beam systems	High geometric precision in clean visibility conditions
Vision (camera)	Sensitive to lighting and adverse weather	Algorithm dependent	Moderate hardware cost, plus compute/maintenance	Reported false positive rate ∼0.2% in optimized setups [10]
FMCW radar	Better than optical under degraded visibility	No	High-frequency RF front-end and processing complexity	Strong ranging capability; performance depends on waveform bandwidth and scene complexity
94 GHz CW ratio (proposed)	Expected weather advantage from W-band propagation; adverse-weather tests not included here	Yes	Field deployment cost not yet quantified in this study	Scaled laboratory: 0.60 cm mean absolute error (Table 1); field false-alarm metrics pending

## Data Availability

Data are available from the corresponding author upon reasonable request.
